# Does virtual reality increase the efficacy of psychotherapy for young adults with mild-to-moderate depression? A study protocol for a multicenter randomized clinical trial

**DOI:** 10.1186/s13063-021-05809-1

**Published:** 2021-12-13

**Authors:** Adrián Montesano, Joan C. Medina, Clara Paz, Helena García-Mieres, Noelia Niño-Robles, Eugeni García-Grau, Josep Cañete Crespillo, Alejandro García-Gutiérrez, Miquel Alabèrnia-Segura, Guillem Feixas

**Affiliations:** 1 School of Psychology and Educational Sciences, Open University of Catalonia, Barcelona, Spain; 2 School of Psychology and Education, Universidad de Las Américas, Quito, Ecuador; 3 Research and Development Unit, Parc Sanitari Sant Joan de Déu, Sant Boi de Llobregat, Barcelona, Spain; 4 Centro de Investigación Biomédica en Red Salud Mental, Madrid, Spain; 5 Department of Clinical Psychology and Psychobiology, Universitat de Barcelona, Barcelona, Spain; 6 Department of Mental Health, Consorci Sanitari del Maresme, Hospital de Mataró, Mataró, Spain; 7 Department of Clinical Psychology and Psychobiology, The Institute of Neurosciences, Universitat de Barcelona, Barcelona, Spain

**Keywords:** Cognitive behavioral therapy, Personal construct therapy, Prevention, Psychotherapy efficacy, Therapeutic alliance, Repertory grid technique, Self and identity, Therapy engagement, Virtual reality

## Abstract

**Background:**

The improvement of psychological treatments for depression in young adults is a pressing issue highlighted in the literature. Its relevance is determined not only because young adults are underrepresented in research, but also to prevent chronic severe mental health disorders later in life. Engagement is considered a key factor for a good therapeutic outcome, especially among young patients. In this sense, virtual reality could be particularly suited to engage young adults in the therapy process. This project aims to improve the psychological treatment of mild-to-moderate depression in young adults by testing out the efficacy of virtual reality-enhanced personal construct therapy (PCT-VR), as compared to personal construct therapy alone (PCT) and to the reference standard cognitive behavioral therapy (CBT). In contrast to CBT, PCT neither educates patients about depression nor gives them directions on the changes to be made in their dysfunctional behaviors or cognitions. Rather, PCT explores the coherence (or conflicts) of thoughts and behaviors with respect to the person’s sense of identity and focuses on meaning-making processes.

**Methods:**

The efficacy of this innovative intervention (PCT-VR) will be compared to PCT and to CBT in a randomized clinical trial. The study includes an appraisal of therapists’ adherence and independent assessments to preserve internal validity. The Beck Depression Inventory-II is the primary outcome measure for calculating both statistical and clinical significance, but other outcomes will also be assessed (e.g., functioning, well-being, anxiety, stress) at pre- and post-therapy and at 6-month follow-up. The trial will be conducted in a naturalistic context, mostly at the usual health care center of each patient. A sample of 225 participants is targeted to reach enough statistical power to accomplish the goals of the study.

**Discussion:**

We expect that providing evidence for PCT-VR will widen the repertoire of evidence-based technology-based psychotherapeutic interventions for young adults and contribute to the prevention of deteriorating courses of the disorder.

**Trial registration:**

ClinicalTrials.gov NCT04321525. Registered on 18 February 2020

## Administrative information

Note: the numbers in curly brackets in this protocol refer to SPIRIT checklist item numbers. The order of the items has been modified to group similar items (see http://www.equator-network.org/reporting-guidelines/spirit-2013-statement-defining-standard-protocol-items-for-clinical-trials/)

**Table:** 

Title {1}	Does virtual reality increase the efficacy of psychotherapy for young adults with mild-to-moderate depression? A study protocol for a multicenter randomized clinical trial
Trial registration {2a and 2b}.	NCT04321525 [ClinicalTrials.gov] [registered before start of inclusion; 18-02-2020]. All the items from the WHO trial registration data set can be found in the body of this protocol.
Protocol version {3}	Version 2.0 17th February 2021
Funding {4}	This research project is partially funded by the Ministry of Science and Innovation (ref. RTI2018-094294-B-I00)
Author details {5a}	A. Montesano, School of Psychology and Educational Sciences, Open University of Catalonia, Spain; J.C. Medina, School of Psychology and Educational Sciences, Open University of Catalonia, Spain; C. Paz, School of Psychology and Education, *Universidad de Las Américas*, Ecuador; H. García-Mieres, Research and Development Unit, *Parc Sanitari Sant Joan de Déu*, Sant Boi de Llobregat, Barcelona, Spain, *Centro de Investigación Biomédica en Red Salud Mental*, Madrid, Spain; N. Niño-Robles, Department of Clinical Psychology and Psychobiology, *Universitat de Barcelona*, Barcelona, Spain; E. García-Grau, Department of Clinical Psychology and Psychobiology, *Universitat de Barcelona*, Barcelona, Spain; J. Cañete Crespillo, Department of Psychiatry, *Hospital de Mataró*, Mataró, Spain; A. García-Gutiérrez, Department of Clinical Psychology and Psychobiology, Universitat de Barcelona, Barcelona, Spain; Miquel Alabèrnia-Segura, Department of Clinical Psychology and Psychobiology, Universitat de Barcelona, Barcelona, Spain; Guillem Feixas, Department of Clinical Psychology and Psychobiology, The Institute of Neurosciences, Universitat de Barcelona, Barcelona, Spain.
Name and contact information for the trial sponsor {5b}	Universitat de Barcelona Passeig Vall d’Hebron, 171, 08035 Barcelona, Spain gfeixas@ub.edu
Role of sponsor {5c}	This is a researcher-initiated clinical trial. Therefore, the funders played no role in the design of the study and will play no part in the collection, analysis, and interpretation of the data and in writing the manuscript.

## Introduction

### Background and rationale {6a}

In depressive disorders, the most common severity level is mild to moderate, accounting for up to 81% of all cases [1]. In turn, the prevalence of mild depression has been estimated to range from 10.6 to 14.8%, while the prevalence estimation for moderate depression is 4.52% [2]. Even when the severity is not high, mild-to-moderate depression bears important clinical implications, as reflected in the fact that the number of publications (Scopus) on this term has tripled in the last 10 years. On the one hand, an episode of this kind may be the onset of a series of depressive periods with a high risk of chronicity. On the other hand, the way a mild-to-moderate episode is resolved may affect the future incidence of major depressive disorder. Certainly, there is enough evidence proving that the incidence of recurrent major depression is more common in patients who have had a previous depressive episode, either mild or moderate. These implications can be especially relevant in young adults that have depression for the first time. In a study [3] with 7199 Danish patients who attended the hospital and met the diagnosis for depressive episode according to ICD-10, 15% was of mild and 44% of moderate intensity. At a 6-year follow-up, 28% of those with mild and 30% of those with moderate intensity were inpatients again, most of them with a diagnosis of recurrent depression but also for other problems that ranged from anxiety to bipolar disorder.

One of the strategies to reduce the impact of depression is the early screening and effective treatment of the first symptoms. It has been proved that the earlier people suffer depression in their lives, the worst their evolution is in terms of recurrence, comorbidity, and other complications. Zhu et al. [4] emphasized the need to provide care to young people and the young adult population as a high priority, in order to prevent the serious consequences resulting from suffering major depression in areas such as educational, personal, and social development, as well as satisfaction with work/study and relationships [5].

Several clinical guidelines point out that psychotherapy is the first-line treatment to address mild-to-moderate depression in teenagers and adults, and pharmacological treatments are reserved to more severe and complex cases that do not respond to psychotherapy [6]. Research about different treatments for depression highlights that psychological therapies are effective and contribute to preventing more severe and recurrent depressive disorders [7]. Regarding the theoretical orientation of the psychological treatments applied, the literature shows that cognitive behavioral therapy (CBT) is the approach that has published more studies, obtaining thus more prestige [8], whereas others claim that its efficacy is diminishing [9]. Indeed, current evidence is not conclusive about signaling one particular form of psychotherapy as clearly more effective than the others, and some reviews consider also other well-established psychological treatments [10], there is a lot of room for improvements (i.e., more than 40% do not respond, only 30% achieve a complete recovery, and relapse rates are high).

On the other hand, some research has pointed out some other factors than therapeutic orientation. Khan et al. [11] claimed that getting the patients engaged in an active therapeutic program was crucial, especially if we consider a dropout rate of 35% [12]. As the engagement in therapy relies on the preferences and attitudes of each patient, therapists might benefit from the availability of a variety of evidence-based psychotherapeutic alternatives. Along these lines, Cuijpers et al. [13] emphasized the importance of personalizing therapies. Another factor that supports the need for adapting the treatment to the patient’s preferences and characteristics is the therapeutic alliance (including the agreement on the therapy tasks), acting as the best predictor of success in virtually any therapy modality [14].

Personal construct therapy (PCT) [15, 16] is based on an idiographic approach in which treatment is tailored to the individual, working collaboratively with their constructions of their identity and of the people around them. It is assumed that each person construes (interprets) the world in their own unique way based upon their system of personal constructs (dimensions of meaning) [17]. PCT has experienced in recent decades a broad theoretical, clinical, and research development [18]. It has also obtained positive evidence in various meta-analyses [19, 20], achieving better results than the waiting list and some common treatments and equivalent results to other active control treatments. At follow-up, patients who received PCT tended to do better than controls. Given that PCT is particularly suited to fit the personal values and attitudes of each patient, it might facilitate the personalization and engagement of young adults facing psychological problems.

Based on PCT, the repertory grid technique (RGT) [21, 22] has been used in at least 3000 publications [23]. It assesses self-concept and cognitive structure from the person’s descriptions of themselves, what they consider to be their “ideal self,” and others. Despite its idiographic emphasis, the RGT allows the derivation of a series of indexes for the study of patient groups, including those diagnosed with depression [24]. These patients have been found to have a high discrepancy between the views of the actual and ideal self (i.e., low self-esteem), and a high perceived dissimilarity between the self and others (i.e., high perceived social isolation/high perceived sense of being different to others). They have also shown many implicative dilemmas, a type of cognitive conflict flagging that the person associates the symptom with positive characteristics of their identity [25]. Such dilemmas indicate that elimination of the symptom, although at one level is desired, may be associated with an expectation of undesirable changes in the person’s identity, thus providing an explanation for resistance to change and relapse. The results of the RGT allow interventions to be tailored to such aspects of the patient’s construing, focusing on targets such as the resolution of dilemmas [26]. This dilemma-focused intervention has been found to contribute with a large effect size (*d* = 1.13) in the reduction of BDI-II scores in a randomized clinical trial with 128 depressive patients. Thus, PCT is a promising treatment for depression that needs further evidence.

In recent decades, several interventions with virtual reality (VR) have been created in the field of personal change and psychotherapy [27, 28], but there are few focused on people with depression. A recent meta-analysis [29] found that VR-based therapies were more effective than controls at post-test for anxiety, although no conclusions could be reached in relation to depression since there were only a few studies, with very limited sample sizes. Promising results were obtained by Falcone et al. [30] using self-identification with a virtual body within immersive VR; the intervention reduced self-criticism and increased self-compassion in a small sample of depressed patients. They suggested that VR has considerable clinical potential and encourage the development of new methods to be tested in controlled trials. In our view, the incorporation of VR interventions in psychological treatments could arouse, at least in young patients with a preference for new technologies, a great interest and facilitate their engagement with the treatment.

In this research project, we examine the efficacy of a VR app named Explore Your Meanings (EYME), based on PCT, that allows for the tailored immersive exploration of personal meanings and self-identity as part of a psychological treatment for depression. EYME immerses patients in a virtual 3D scenario where they can visualize their personal meanings and their relationship with their most significant people, derived from the analysis of the data generated with the RGT. During the exploration of such personalized scenario, therapy objectives and (ambivalence towards) change are addressed with the assistance of a psychotherapist.

Thus, this project will allow us to test the hypothesis that VR-enhanced personal construct therapy (PCT-VR) will be more efficacious in the treatment of mild-to-moderate depression of young adults than PCT alone and the well-established CBT. We expect that PCT-VR, based on the immersive exploration of the patient’s self-identity, will make the treatment more attractive for young adults, increasing their engagement in the therapy process; therefore, our hypothesis is that PCT-VR will result in a greater reduction of depressive symptoms (primary outcome) and improve psychological functioning, well-being, and satisfaction, compared to psychotherapies delivered in the usual format. As a secondary hypothesis, we expect that PCT, thanks to its personalized and non-prescriptive nature, will be more efficacious than CBT. Also, as reflected in the literature, we expect that the quality of the therapeutic alliance will be associated to the outcome, but we will also explore whether this association varies in its intensity across therapy modalities.

### Objectives {7}

With this project, we pursue the goal of increasing the efficacy of psychological therapies for mild-to-moderate depression in young adults. We aim to obtain better clinical results with the promising (PCT) and novel (PCT-VR) treatment modalities than with the currently most prestigious CBT. By obtaining supporting evidence for these therapies, the range of efficacious psychotherapeutic alternatives for depression would be increased, and this would benefit the potential for adapting the interventions to the personal styles, characteristics, and preferences of each patient. An optimal personalization of the treatment favors the patient engagement in therapy and strengthens the therapeutic alliance which, in turn, should be reflected in the facilitation of the change process.

#### Specific objectives

1. To assess the efficacy of an innovative VR-enhanced treatment modality of psychotherapy (PCT-VR) which has never been tested before. Differential outcomes will be considered not only in terms of statistical but also clinical significance and remission rates. In addition to the reduction of depressive symptoms, the change in psychological functioning, well-being, and satisfaction with the services provided will be assessed.
2. To evaluate the efficacy of PCT for mild-to-moderate depression in young adults, a psychotherapeutic approach with some supporting evidence for a variety of disorders and conditions but still insufficient to be considered efficacious for depression.
3. To verify the good levels of efficacy shown by CBT in the treatment of depression. This might be relevant in the light of the controversy about its efficacy in recent times [9].
4. To gauge the contribution of the therapeutic alliance in the outcomes of the three different treatments being tested. The rationale for focusing on this variable relies on the fact that, as mentioned above, the therapeutic alliance is the best predictor of success across treatment modalities [31]. In addition, engagement (which is a factor of the therapeutic alliance) is purported to be crucial in the treatment of young adults and targeted by the nature of two treatment modalities being tested out in this trial (PCT and PCT-VR).

### Trial design {8}

This is a multi-center superiority randomized clinical trial, comparing the efficacy of two novel brief treatment modalities, PCT and PCT-VR, to CBT, a well-established treatment, with respect to their efficacy in treating young adults with mild-to-moderate depression. The comparison between PCT and PCT-VR will reveal the differential efficacy of including a VR app in the latter condition. The patient allocation ratio is 1:1:1.

## Methods: participants, interventions, and outcomes

### Study setting {9}

This multi-center study will be conducted at several health facilities (primary care centers, tertiary hospitals, university clinics) in Barcelona and surrounding areas. Two mental health centers have confirmed their participation in the study (Hospital de Mataró and CSM Nou Barris). Due to COVID-19 restrictions and heavy workload, primary care centers that showed initial interest in participating in the study are not included at the time of writing this article. However, if restrictions change while the trial is carried out, these centers accepted the invitation to participate. An updated list of the participating centers will be updated in ClinicalTrials.gov.

Patients will be recruited at each health center and from an open call through social media. Patients are considered for inclusion if they meet the criteria defined below.

### Eligibility criteria {10}

#### Inclusion criteria

All participants will meet the diagnostic criteria for mild or moderate depressive episode according to ICD-10 (current version in the Catalan health system) diagnosed using the MINI International Neuropsychiatric Interview (MINI), and a score above 13 and below 29 on the Beck Depression Inventory-II (BDI-II) (see the “Outcomes” section). The age range is established between 18 and 29 years inclusive.

#### Exclusion criteria

Patients presenting bipolar or other affective disorders (ICD-10), psychotic symptoms, substance abuse, organic brain dysfunction, acute suicidal ideation, or intellectual disability will be excluded from the study. Those who are receiving psychological treatment will not be accepted either, unless it is suspended at the time of inclusion in the study, in agreement with patients and their therapists. The presence of other comorbid conditions (anxiety, eating or personality disorders, etc.) and antidepressant medication treatment will not be causes for exclusion, but they will be assessed and recorded, and their influence will be explored statistically. Finally, individuals who do not have enough competence to communicate in Spanish or Catalan, or patients with substantial visual, hearing, and cognitive deficits, will not be included.

### Who will take informed consent? {26a}

The trial protocol will be presented to medical professionals of the health facilities participating in the study to stimulate the recruitment of patients according to the eligibility criteria. Those patients interested in participating will be asked to authorize professionals to provide the information that enables a research assistant to contact them. After referral or response to social media calls, potential participants will be contacted by the trial coordinator to arrange a date for the initial assessment, which will be conducted by psychology graduate research assistants. In that first contact, the characteristics of the study will be explained to the referred patients who will be invited to discuss any remaining questions and to sign the informed consent if they agree to participate.

### Additional consent provisions for collection and use of participant data and biological specimens {26b}

The use of the data for ancillary studies, not covered by the objectives of the main trial, will be evaluated by the Institutional Review Board of the *Universitat de Barcelona*. If the ancillary study is approved and the informed consent does not cover the new proposed use of the data, additional signed consent will be offered to each participant included in that study.

### Interventions

#### Intervention description {11a}

Specific therapy protocols for the three treatment conditions of the study (CBT, PCT, and PCT-VR) have been developed. These three treatments will follow the same format: a maximum of 10 individual 1-h sessions, with one or two booster sessions at about 3 months after the tenth session. The protocols are not standardized since they are based on personalized case conceptualization. In both PCT and PCT-VR, the construction of self and others will be explored with the RGT; but while in the former, only 2D diagrams will be used (paper); in the latter, this will be done with EYME, in an immersive VR environment for each patient. A video with a brief illustration of the use of EYME in the treatment can be found at https://www.youtube.com/watch?v=aGOOo8p7EfM. Also, a brief description of the RGT procedure which will be applied in these two treatment conditions follows.

The RGT aims to explore the constructions with which a person represents the interpersonal world in her or his own terms, and it is administered in a semi-structured, face-to-face interview. First, 10 to 20 elements (self now, significant family members and friends, and ideal self) are obtained. Then, the constructs (bipolar dimensions of meaning) the person uses to categorize them are elicited using similarity and difference questions. Note here that the addition of the Ideal Self element (“how I would like to be”) allows the therapist to grasp the person’s goals as well. Then, these elements and constructs provided by the interviewee are entered into a blank grid form to rate each element (self, others, and ideal self) according to the elicited constructs using a 7-point scale, thus creating a data matrix that reflects the construct usage of respondents in their interpersonal world. Multivariate statistical analyses of this matrix reveal the patterns of relationships among constructs and the position of self and others in the meaning system of the individuals being assessed, which can be plotted in a two-dimensional space. Also, Euclidian distances and correlations provide a series of psychological indices (e.g., self-ideal discrepancy) indicating structural characteristics of that system. As reflected in the literature, these analyses have allowed researchers and professionals a detailed and personalized understanding of patients’ personal views of themselves and others, which has been used to target specific psychotherapeutic interventions using case formulation [32, 33].

The three treatment modalities of the study will be applied by psychologists with specific clinical experience and training (master’s level or higher) in the therapy modality assigned. Senior therapists with more than 15 years of experience will conduct specialized training and weekly supervision sessions with the therapists. Indeed, to control for researcher allegiance and to promote high treatment quality in the three arms, the research team involved in the study included senior members with extensive experience in each of the treatment conditions being tested (see the “Authors’ information” section). The treatment will be provided in the health centers in which participants are being recruited.

The PCT therapy protocol will be based on the case conceptualization as described by Winter and Procter [34] and on a series of texts describing the applied form of PCT [16, 25, 33] as well as on previous literature on PCT for adult depressive patients [35]. These include detailed objectives and tasks for each session that are summarized here.

In the first session, patients are welcomed, their complaints are clarified, and their constructions about themselves and the problems they face are explored (including attributions and possible solutions). In the second session, the RGT is administered, and in the third session, some of the results of the grid analysis are discussed with the patient in an effort to reach an enriched view of themselves and their problem(s). Any diagram used for this exploration of identity will be two-dimensional (2D). Sessions 4 to 9 will be focused on the different aspects of the construct system which need further exploration and reconstruction (cognitive conflicts, construction of “ideal self,” construct differentiation, constriction, family constructs, etc.). As needed, according to the details of each particular case, changes occurring in therapy will be related to the construction of self and others reflected in the results of the RGT. At the end of the ninth session, patients will be asked to rate again the grid elements with the same personal constructs elicited in session 2. In the tenth session, results of this end-of-therapy grid will be visualized and discussed, along with future prospects and elaboration of their personal projects.

The PCT-VR therapy protocol will be identical to the one just described with the exception of the sessions (mainly 3 and 10) in which the constructions of self and others derived from the grid analysis are visualized and explored with the VR application EYME, instead of 2D displays. The head-mounted display used is either Oculus Go or Oculus Quest.

#### Explanation for the choice of comparators {6b}

Metanalyses on treatments for depression point out that psychological therapies are effective [7] and that CBT is the approach that has received more empirical support so far [8]. Therefore, this approach is chosen as the reference standard comparator. In the present study, a CBT protocol was developed with the objective to fit with the number of sessions of the experimental treatment conditions. This protocol is not standardized but based on case conceptualization following the guidelines of the well-known manual of Beck et al. [36] with the updates of Fenell [37] and Beck [38].

#### Criteria for discontinuing or modifying allocated interventions {11b}

Patients will be informed of their freedom to leave the study at any time, for any reason, and without any consequences. The patient data that have been collected up to that moment will be included in the analysis (intention-to-treat approach), unless they require the research team to delete it. Reassignment of patients between the study arms is not contemplated. As an exception, if one patient who is assigned to the PCT-VR arm rejects using VR, then it would be randomized to one of the other two conditions. For those declared as eligible, no harmful effects have been identified so far for the psychological treatments included in the study.

#### Strategies to improve adherence to interventions {11c}

Patient’s treatment compliance will be monitored by attendance to therapy sessions and homework completion. The schedule of therapy sessions from all arms can be adjusted to the individual patient’s needs in order to facilitate therapy completion. Treatment adherence will be monitored by the specialized therapist delivering the treatment, in close contact with the research team.

On the other hand, with regard to treatment integrity and therapists’ adherence to the type of therapy delivered, different strategies will be implemented. Before the start of treatment, differentiated training workshops will be developed for each type of treatment to provide therapists with specific training in the protocols for the therapy formats used in the study. Within the trial, therapy sessions will be audio recorded (as reflected in the informed consent) for facilitating session-by-session supervision with senior members of the team. They will evaluate the performance of the therapists in order to maximize their adherence to the relevant manual and ensure that therapy is beneficial for its recipients. Finally, from the therapy protocols of the three conditions of the study, a scale will be developed to assess the adherence of the therapists to the corresponding protocol. For the CBT arm of the study, therapist adherence will be assessed using the revised cognitive therapy scale [39]. Because there are no pre-existing adherence or fidelity measures for PCT, a scale will be created to measure therapist adherence to this therapy arm. Two trained clinical graduate students blinded to the treatment conditions and trained to use the scale reliably will rate the audiotapes of 10 sessions of CBT, PCT, and PCT-VR. Inter-rater reliability and success at discriminating between the content of the CBT and PCT will be computed.

#### Relevant concomitant care permitted or prohibited during the trial {11d}

Concomitant psychotherapy is not permitted while participating in the intervention phase of the study. Antidepressant and other kinds of psychotropic medication are permitted during the trial, but the dosage will be recorded at baseline, end of therapy, and follow-up assessments.

#### Provisions for post-trial care {30}

No provisions for post-trial care are anticipated.

### Outcomes {12}

#### Primary outcome

The severity of depression will be assessed using the Beck Depression Inventory-Second Edition II (BDI-II) [40]. It is a 21-item self-report with good psychometric properties and acceptability in both the original and the Spanish versions [41]. The total score of the BDI-II is calculated by summing the ratings of the 23 items, resulting in scores ranging from 0 to 63. The mean of the total scores will be compared as the change from baseline to post-treatment and 6-month follow-up assessments to verify the statistical significance. To examine the clinical significance, the proportion of patients who attain clinically significant change according to Jacobson and Truax’s criteria, explained in the data analysis section, will be compared between treatment conditions.

#### Secondary outcomes

General psychological distress will be assessed with the Clinical Outcomes in Routine Evaluation-Outcome Measure (CORE-OM) [42]. It is a 34-item self-report questionnaire for the assessment of subjective well-being, symptoms or problems, life functioning, and risk. It showed good psychometric properties which were preserved when Spanish adaptation was tested with a local sample [43]. This version will be used to assess the change in this study. The mean of the total scores will be compared as the change from baseline to post-treatment and 6-month follow-up assessments to test the statical significance. To measure the clinical significance, the same methods proposed for the BDI-II will be used. The short version (CORE-SFB) of 18 items will be used for session-to-session monitoring of the therapy process.

Symptoms of depression, anxiety, and stress will be evaluated with the Depression, Anxiety, and Stress Scales (DASS-21) [44]. This 21-item version of the DASS comprises seven items for each scale (depression, anxiety, and stress). The scores for each scale range from 0 to 21. There have been validation studies with the Spanish population finding satisfactory psychometric properties [45]. The main advantage of using the DASS-21 as a secondary measure is that it includes the assessment of other symptoms related to, but not equal to, depression. While anxiety and stress are expected to decrease with intervention, the primary outcome and the CORE-OM do not include specific items to assess stress and anxiety. The mean of the total scores for each scale will be compared as a change from baseline to post-treatment and 6-month follow-up assessments to verify statical significance. To gauge clinical significance, the same methods proposed for the BDI-II will be used.

The quality of the therapeutic alliance will be assessed with the Session Rating Scale 3.0 (SRS 3.0) [46]. It is composed of 4 items answered through a visual analog scale of 10 cm. They measure clients’ appreciation of the relationship with their therapist, agreement about the goals and issues discussed in the session, agreement with the method or approach, and global assessment of the session. The Spanish version obtained good levels of validity and reliability [47]. This measure will be used in each session, and its association with treatment outcome and modality will be analyzed. The mean of the total scores will be compared between treatment conditions for each session.

For diagnosing psychiatric disorders according to ICD-10, the Mini International Neuropsychiatric Interview (MINI) [48] will be applied. In this study, MINI is used for diagnosing inclusion (mild and moderate depression) and exclusion (major depression disorder, bipolar disorders, psychotic symptoms, etc.) criteria, as well as comorbid conditions (e.g., anxiety, eating, or personality disorders). Evaluators will conduct the MINI-based clinical interview before allocation and after treatment, and percentage of remitted patients will be compared across treatment conditions.

### Other measures

#### Consumer Reports Effectiveness Scale (CRES-4) [49]

It consists of four items designed to evaluate whether patients are satisfied with the therapy they have received and if it has been perceived as effective or not. The total score is intended to reflect the degree of satisfaction with the treatment received. The mean of the total scores for each treatment condition as assessed at follow-up will be compared.

#### Life Satisfaction Scale [50]

The patient is asked to rate on a Likert scale of 10 points the overall degree of satisfaction with life at the present moment. This single item has shown to have an acceptable reliability, and it is theoretically consistent. The mean of the total scores will be compared across treatment conditions as a change from baseline to post-treatment and 6-month follow-up assessments.

#### Client change interview [51]

This semi-structured interview was created to assess the changes produced throughout therapy, the useful and adverse effects of interventions from the patient’s perspective. Patients are asked about the positive and negative changes they have experienced with the intervention. Questions are also asked about the attribution of such changes, and their probability of occurrence without intervention. It is administered after completion (post-treatment), and its function is to identify both changes that patients are aware of (in their own words) and adverse effects (too often neglected in psychotherapy research). All these aspects might go unnoticed with standardized questionnaires.

#### Participant timeline {13}

**Fig. 1:**
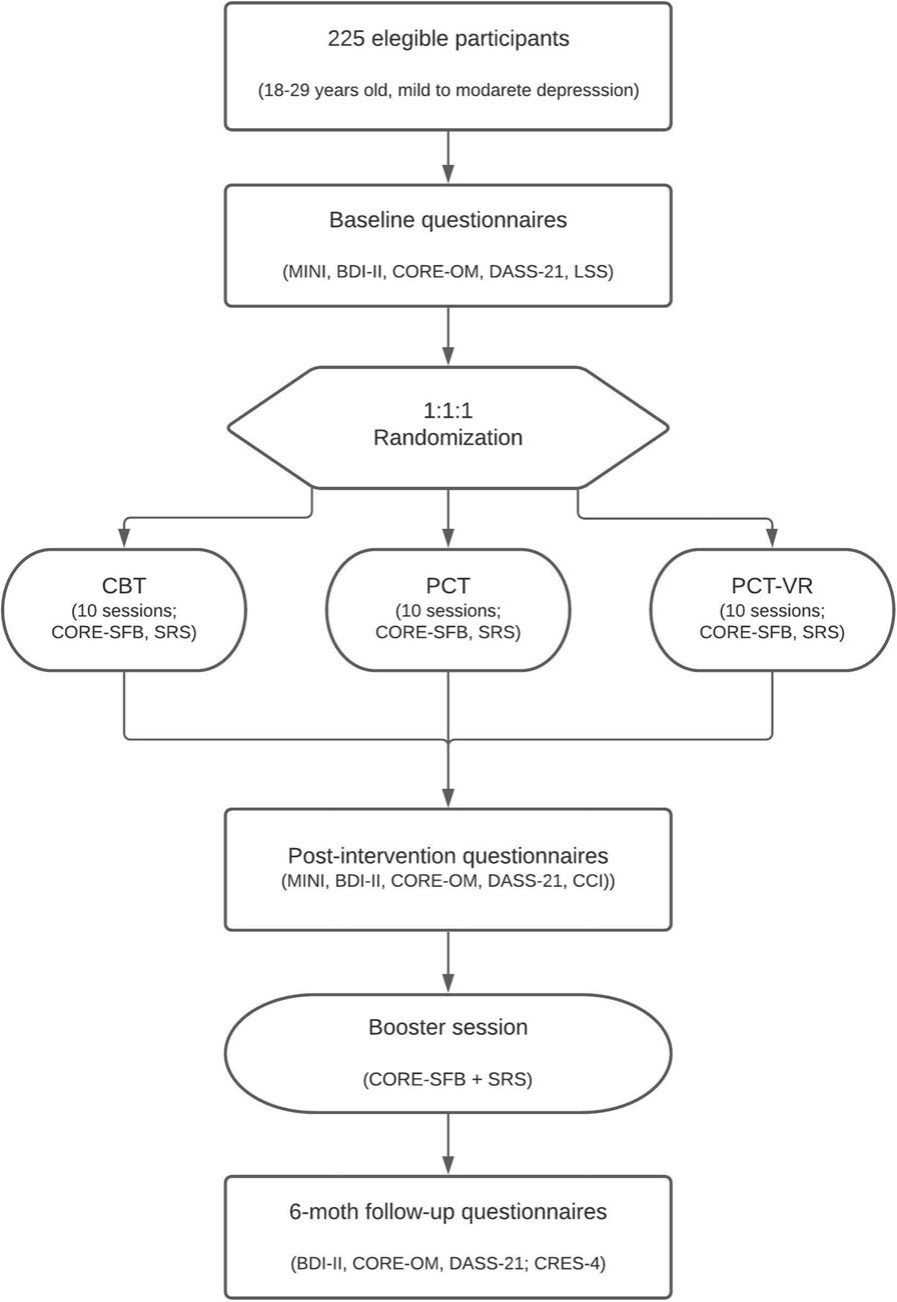
Participant timeline. MINI, International Neuropsychiatric Interview; BDI-II, Beck Depression Inventory second edition; DASS-21, Depression, Anxiety, and Stress Scale; CORE-OM, Clinical Outcomes in Routine Evaluation-Outcome Measure; CORE-SFB, Clinical Outcomes in Routine Evaluation-Short Form Version B; SRS, Session Rating Scale; LSS, Life Satisfaction Scale; CBT, cognitive behavioral therapy; PCT, personal construct therapy; PCT-RV, personal construct therapy virtual reality; CCI, Client Change Interview; CRES-4, Consumer Reports’ Effectiveness Scale

Table 1 and Fig. 1 show the scheduling of evaluations and the participant timeline, respectively.

**Table 1:** Schedule of assessments

	Pre-randomization	Post-randomization		
	Screening/baseline	Intervention (every session)	Post-treatment	6-month follow-up
SCV	X			
MINI	X		X	
BDI-II	X		X	X
DASS-21	X		X	X
CORE-OM	X		X	X
CORE-SFB		X		
SRS		X		
CRES-4				X
LSS	X			X
CCI			X	

*Abbreviations*: *SCV*, sociodemographic and clinical variables; *MINI*, International Neuropsychiatric Interview, *BDI-II*, Beck Depression Inventory second edition; *DASS-21*, Depression, Anxiety, and Stress Scale; *CORE-OM*, Clinical Outcomes in Routine Evaluation-Outcome Measure; *CORE-SFB*, Clinical Outcomes in Routine Evaluation-Short Form Version; *SRS*, Session Rating Scale; *CRES-4*, Consumer Reports Effectiveness Scale; *LSS*, Life Satisfaction Scale; *CCI*, Client Change Interview

### Sample size {14}

The effect sizes reported in the literature about the differential efficacy of psychotherapy for depression are usually small [52]. Besides, the severity of the disorder of the targeted population is low (mild-to-moderate depression). Therefore, we anticipated a small effect size and selected 0.45 as the parameter for sample size calculation. The statistical power considered for the sample size calculation was 0.80, with a dropout rate of 5%, and an *α* significance level of 0.1 (one-sided). A total of 225 participants, 75 patients per arm, are required to detect a small effect size in our primary outcome measure (BDI-II).

### Recruitment {15}

Participants will be recruited from several health services in Barcelona and surrounding areas which already have agreements with the *Universitat de Barcelona*. In addition, we will disseminate an open call through social networks.

## Assignment of interventions: allocation

### Sequence generation {16a}

Participants will be randomized to one of three arms in a 1:1:1 ratio, stratified by health center. Assignment will be conducted with computer-generated random block sizes of 3 or 6, masked from the study staff.

### Concealment mechanism {16b}

The allocation sequence will be concealed from the researcher enrolling and assessing participants in sequentially numbered, opaque, sealed, and stapled envelopes. The aluminum foil inside the envelope will be used to render the envelope impermeable to intense light. Corresponding envelopes will be opened only after the enrolled participants completed all baseline assessments and it is time to allocate the intervention.

### Implementation {16c}

Randomization will be performed by a staff member of the Department of Clinical Psychology and Psychobiology of the *Universitat de Barcelona* completely blind to the study and to treatment conditions and with no clinical involvement in the trial. The trial coordinator will save the sequentially numbered, opaque, sealed, and stapled envelopes. Allocation will be made after the research assistant obtains the patient’s consent during the baseline assessment.

## Assignment of interventions: blinding

### Who will be blinded {17a}

Researchers, patients, and therapists will not be blinded since this is impossible due to the major difference between the groups (use of virtual reality in PCT-VR). Moreover, therapists and (senior) researchers will need to follow a manual related to the corresponding arm of the study. However, blinding to study hypotheses will be fostered by explaining to all patients and therapists that the study objective is to assess the efficacy of psychotherapy for young adults, regardless of the type of treatment. On the other hand, researchers conducting outcome assessments will be blind to treatment allocation.

### Procedure for unblinding if needed {17b}

There is no prevision that unblinding will be needed

## Data collection and management

### Plans for assessment and collection of outcomes {18a}

All data from questionnaires will be gathered using an automatized system (e.g., Qualtrics™), even if participants are physically accompanied by their therapist or evaluators when completing the forms. To ensure data collection in every stage (baseline, therapy monitoring, and follow-ups), a number of quality control procedures will be used, such as providing participants with warnings whenever a question has been left blank to minimize the frequency of missing answers, notifying the research coordinator indicating that a participant is not eligible (e.g., the score in the BDI-II is above 28), and warning the research team if a patient is at risk (e.g., suicidal ideation) or a participant has not completed the questionnaire.

Screening interviews will be conducted in synchronous videoconference settings by psychology graduates specifically trained in the MINI by researchers of the team. Interviewers will enter data in the data system right after the interview whereas participants will be emailed individualized links to complete baseline questionnaires (BDI-II, CORE-OM, and DASS-21). All diagnoses will be supervised by the trial coordinator to ensure validity and homogeneity throughout the recruitment process. Then, to monitor the psychotherapy process, participants will also be emailed individualized links to complete the CORE-SFB just before each session, and the SRS 3.0 for the therapeutic alliance at the end. The post-treatment assessment consists of all the baseline measures, including the MINI interview which will be performed, whenever possible, by the same evaluators who carried out the baseline interview and again supervised by the trial coordinator. In addition to these instruments, the change interview will also be conducted by a researcher who did not have previous contact with the patient. With respect to follow-up measures, participants will be emailed (and reminded by telephone, if necessary) an individualized link to the questionnaires containing the same assessment protocol as in previous time points plus the CRES-4, for evaluating treatment satisfaction.

### Plans to promote participant retention and complete follow-up {18b}

Patients will receive extensive information about the study setup, and the importance of completion of the treatment and the follow-up assessments will be stressed. With respect to psychotherapy, whenever a session is missed, the therapist will contact the patient to re-schedule the session and to identify the reasons for non-attendance to prevent missing future sessions. Patients are allowed to abandon the study at any time. The trial coordinators will inquire about the reasons for withdrawal while respecting their right to do so. In addition, they will be encouraged to complete the follow-up questionnaires. This will be important to the intention-to-treat analyses in an attempt to maximize the number of participants in each study arm. All patients will be reminded throughout the study to fill out the questionnaires by individualized emails as well as by therapists and outcome assessors. Throughout the follow-up period, the trial coordinator will check the responses and, if necessary, contact the patients for completion of their follow-up.

### Data management {19}

Data derived from questionnaires will be gathered through a secure web-based platform following security guidelines and policies and that has been approved by the university’s local ethical committee. The research team carrying out (outcome assessors) and supervising (trial coordinator) the assessment process and promoting data quality will undergo a 4-h training session on the data system and will count on the continuous supervision of principal investigators of the trial. Back-ups on the protected research server will be made regularly.

### Confidentiality {27}

With regard to the specific procedures to handle data, all researchers involved in its collection, custody, storage, and analysis within this trial will keep the strictest confidentiality about the personal information of participants. Likewise, the researchers who are part of the present project will commit themselves to not make any use of the personal data to which they may have access in the exercise of their assigned functions for any purpose unrelated to the project, as well as to neither share nor reveal that information to any third party. This obligation extends beyond the expiration of the research project and will be formalized by signing a confidentiality statement. The personal data collected in the context of the present research (recordings of the therapies, homework, etc.) will be stored securely in locked closets for both paper and digital (encrypted hard drive) formats. Only the principal investigator of the project may grant access to such files. For data analyses, anonymized databases will be used to further protect participants’ identities, assigning them an alphanumeric code. In any case, all the members of the trial will adjust to the provisions of the European law on the protection of personal data.

### Plans for collection, laboratory evaluation, and storage of biological specimens for genetic or molecular analysis in this trial/future use {33}

Not applicable since biological specimens are not collected for this trial.

## Statistical methods

### Statistical methods for primary and secondary outcomes {20a}

Intention-to-treat analyses (i.e., using outcomes from all randomized participants) will be performed for the main outcomes. Linear mixed models will be used to analyze and to compare treatment effects for the three treatment conditions (CBT, PCT, and PCT-VR). Baseline measurements, gender (coded as woman/male/not-binary), age, and clinical variables will be entered in the model as covariates. Pairwise comparisons will be used to test the hypothesis that PCT will lead to better outcomes than CBT and that PCT-VR will result in better outcomes than PCT. For all the analyses, effect sizes will be calculated.

In addition to the statistical significance, the proportion of patients who attain clinically significant improvement on the outcome measures will be calculated and compared between treatment conditions. The clinically significant improvement will be determined considering the proposal of Jacobson and Truax. For them, two conditions must be met: reliable change (reliability of pre-to-post difference in scores not being produced by chance) and clinically significant change (post-treatment scores lying in the normal instead of the clinical range). To test the differences in frequencies between the treatment conditions, a chi-square test will be applied.

### Interim analyses {21b}

There will be no interim analyses regarding the main objective of the trial. However, secondary or complementary analyses (e.g., correlational) of the baseline data can be performed.

### Methods for additional analyses (e.g., subgroup analyses) {20b}

The inclusion of CORE-OM will permit to assess the degree of therapeutic change with regard to psychological distress, psychosocial functioning, and suicide risk. Also, the application of CORE-SFB and SRS 3.0 in each session will allow to assess the change experienced in psychological distress and therapeutic alliance as a function of the number of sessions and the percentage of change session by session. We will conduct growth mixture modeling based on these weekly assessments to identify distinctive patterns of change and evolution of the therapeutic alliance. If several patterns can be identified, we will perform mixed linear models and repeated-measure analyses of variance to compare patients’ characteristics in each pattern, and we will identify possible associations between change in psychological distress and therapeutic alliance scores. Then, we will consider using multinomial logistic regression to compute predictive models for the patterns from patients’ characteristics. Also, we could use hierarchical linear regression to establish the power of each pattern to predict treatment outcomes. Subgroup analyses by gender will be explored to check if there are significant differences in the therapy course. Secondary analyses will explore the potential mediator or moderator role of the cognitive measures obtained by the RGT, especially those measures related with cognitive conflicts. Finally, if the results of the trial support our main hypotheses, a complementary protocol for qualitative analyses of therapy sessions’ verbatim will be issued in due time with the goal of exploring the change mechanisms.

### Methods in analysis to handle protocol non-adherence and any statistical methods to handle missing data {20c}

The analysis will follow an intention-to-treat strategy. As missing values will be lessened by Qualtrics quality control strategies, no imputation plan is considered in advance.

### Plans to give access to the full protocol, participant-level data, and statistical code {31c}

Non-identifiable datasets can be made available by the principal investigators upon reasonable request and in agreement with the research collaboration and data transfer guidelines of the University of Barcelona. The current document outlines the full protocol.

### Oversight and monitoring

#### Composition of the coordinating center and trial steering committee {5d}

This is a multi-center study coordinated by the University of Barcelona in collaboration with the Open University of Catalonia. The main trial team is formed by two principal investigators (AM, GF) overseeing all aspects of the study, training of staff, and taking supervision of the clinical development of the interventions. They will meet weekly to monitor the progress with specific subgroups of the research team and with the trial coordinator. The latter, in coordination with the other members of the research team, will manage participant recruitment and treatment allocations, organize data entry, and monitor safeguarding data quality.

#### Composition of the data monitoring committee, its role, and reporting structure {21a}

An independent data monitoring committee was not deemed necessary given that this is a trial focused on behavior, and adverse events, if any, are expected to be minimal.

#### Adverse event reporting and harms {22}

As indicated previously, adverse events, if any, are expected to be minor due to the nature of the interventions. Regarding the intervention with VR, measures will be taken to prevent the occurrence of side effects arising from the use of VR devices that can be annoying or temporarily affect normal activity. Under certain conditions, the use of these devices can produce mild discomfort related to dizziness, nausea, headache, or eye dryness in some users. These discomforts can arise at any time throughout and beyond the therapy sessions. Currently, the factors that increase the risk of these unwanted reactions are known [53], and we will take measures to prevent them:

1. The virtual environments will be designed to be stable so that any movements will arise only from the user’s action.
2. These movements will be reduced to a minimum by using techniques such as teleportation, which reduces the side effects of the simulation to almost zero.
3. The participants will be instructed to immediately report any side effects to the therapist, who will interrupt the session until the complete disappearance of any nuisance.
4. At the end of each session, participants should remain seated for a few minutes until it is clear that side effects are not present. If there is any discomfort, even to a small degree, they will remain seated, accompanied by the therapist until their complete recovery.

Apart from this VR-related dizziness, no harmful effects have been identified in the psychological treatments included in the study. However, at the end of each session, the presence and type of adverse events reported by the participants or observed by the investigators will be systematically recorded. The frequency, severity of all the detected harms, and causal relation to the treatment interventions will be analyzed and reported in forthcoming publications.

#### Frequency and plans for auditing trial conduct {23}

No audits have been planned. The main trial team meets weekly (or as needed) to monitor the project progress. Moreover, senior international researchers will oversee the project twice per year, checking the development and completeness of the research plan.

### Plans for communicating important protocol amendments to relevant parties (e.g., trial participants, ethical committees) {25}

All substantial amendments will be submitted to the competent authority (ethics boards involved) for approval and will be notified on trial registries (ClinicalTrials.gov). In case amendments affect participants in any way, they will be informed about the specific changes, and if needed, additional consent (previously approved) will be requested and registered. Non-substantial amendments will be recorded and filed using protocol version and date identifiers.

### Dissemination plans {31a}

The results of this research will be disclosed completely in international peer-reviewed journals and conference presentations. Both positive and negative results will be reported. We will also plan dissemination to the relevant patient and clinical interest groups.

## Discussion

This protocol describes a funded RCT evaluating the efficacy of psychotherapy for depression in young adults. To the best of our knowledge, this is the first RCT collecting evidence for PCT as a psychotherapeutic approach for young adults with depression, which differs in some respects from the well-established CBT. By focusing on identity issues, PCT (regardless of using VR or not) uses alternative therapeutic strategies and techniques [16–20] which, if proven effective, may increase the range of evidence—procedures for the psychotherapies for depression. Furthermore, the capacity of PCT (with and without VR) for patient engagement is expected to influence patient adherence and, eventually, their efficacy over standard treatment. In this sense, psychotherapy might not only better the life of affected young adults and reduce the costs associated with mild-to-moderate depression, but also prevent a deteriorating course which might involve much higher long-term personal, social, and economic costs. Thus, the results from this trial can make a substantial contribution to chronicity prevention [54, 55]. Future long-term follow-up studies could shed light on this relevant issue.

A specific innovation being tested in the PCT-VR arm of this trial is the exploration of the patient’s personal identity in an immersive VR environment, above named EYME. The applications of VR to psychotherapy are now numerous, varied, and reasonably efficacious for many psychological problems [27, 28] but scarce for depression. If the evidence gathered in this study supports it, EYME might become a valuable innovation in the application of VR to improve health and well-being. Besides, with respect to traditional assessment based on standardized questionnaires, EYME is an innovative alternative not only by its use of VR but also by working with the personal constructs of the interviewee as applied to self and significant others. This immersive exploration of self-identity is based on multivariate statistics derived from the analysis of the RGT data. Taken together, these innovative features may add a significant value to existing psychological assessment techniques.

Future developments of PCT-VR with EYME might find it useful as a transdiagnostic instrument to be included in the so-called internalizing disorders that are the leading contributors to health burden among children, adolescents, and young adults globally [56, 57]. As a transdiagnostic approach, it can also be applied to adult clinical problems for which identity issues are central and more effective treatments are needed (e.g., psychosis, personality disorders). Beyond the clinical terrain, the present project has a high potential for transference, since EYME, as a self-identity exploration technique, could be applied in vocational guidance, business consultancy, or other ambits in which decision-making processes are central.

## Trial status

The recruitment of participants of this study is planned for May 2021. The protocol was modified for online delivery of the assessment, and the current version and date are version 2.0 and 17 February 2021, respectively. The trial is scheduled to end in December 2023.

## Data Availability

The present manuscript does not contain any data. However, the explanation of how data, resulting from the present trial, will be shared is explained in the “Plans to give access to the full protocol, participant-level data, and statistical code” section.

## Abbreviations

BDI-II: Beck Depression Inventory, Second Edition
CBT: Cognitive behavioral therapy
CORE-OM: Clinical Outcomes in Routine Evaluation-Outcome Measure
CORE-SFB: Clinical Outcomes in Routine Evaluation, Short Form Version
CRES-4: Consumer Reports Effectiveness Scale
DASS21: Depression, Anxiety, and Stress Scales
EYME: Explore Your Meanings app
ICD-10: International Statistical Classification of Diseases and Related Health Problems, 10th version
MINI: International Neuropsychiatric Interview
PCT-VR: Personal construct therapy-Virtual Reality
PCT: Personal construct therapy
RGT: Repertory grid technique
SRS 3.0: Session Rating Scale version 3.0
VR: Virtual reality
